# Kazakhstan Aims to Reclaim Its Nuclear Legacy with Fusion Energy

**DOI:** 10.1021/acscentsci.5c01960

**Published:** 2025-10-23

**Authors:** Diana Kruzman

## Abstract

Once a testing ground for Soviet weapons, the country is using a unique facility to find materials that withstand plasma 10 times as hot as the sun’s core.

## Graphical abstract

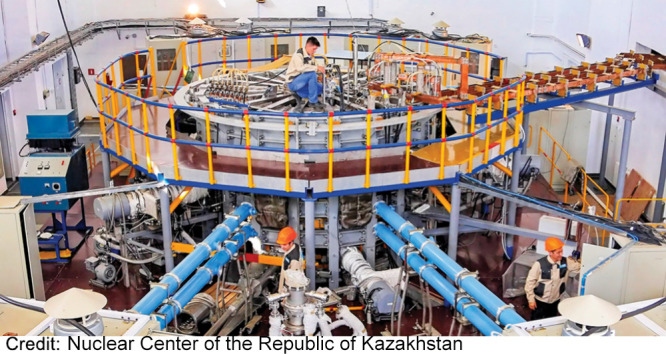

When Baurzhan Chektybayev was a child, the nuclear test site near his home in northeast Kazakhstan was far from the first thing on his mind. Chektybayev was born in 1985, when Kazakhstan was still part of the Soviet Union and used as a proving ground for the country’s nuclear arsenal. Even after the testing stopped and Kazakhstan gained its independence in 1991, the activities of the nuclear scientists based at Kurchatov, a town less than 150 km from Chektybayev’s home city of Semipalatinsk, were shrouded in secrecy.

But as he grew closer to graduating high school and choosing his field of study, Chektybayev started learning more about nuclear science—not for testing weapons but for building reactors to produce energy. Kazakhstan was about to embark on an ambitious new project, one that drew Chektybayev in and has kept him busy in the nearly 20 years since he started his career.

Kurchatov, where weapons that could wipe out humankind were once built, would become a site for the development of nuclear fusion—an energy source that could power the Earth for generations.

**Figure:**
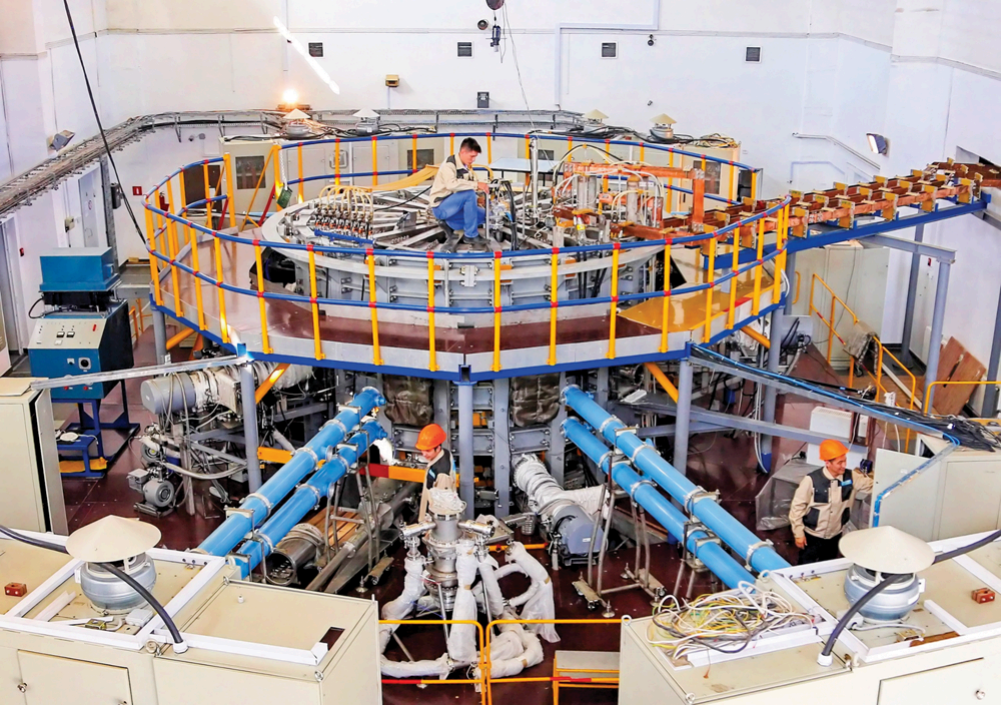
The Kazakhstan tokamak for material testing generated its first plasma in 2019. Credit: National Nuclear Center of the Republic of Kazakhstan.

Unlike fission, the process that powers nuclear reactors today, fusion involves the collision of atomic nuclei. They fuse into new elements, producing massive amounts of energy. It is the same type of process that keeps the sun ablaze.

Notoriously difficult to achieve, the technology is, as the saying goes, “always 30 years away.” Using its unique combination of research expertise and technological capabilities, Kazakhstan’s National Nuclear Center (NNC) is helping to incrementally advance the world toward making nuclear fusion a practical source of energy.

Top of the list for the NNC is discovering materials that are tough enough to be used in a fusion reactor, given the extreme conditions inside. To achieve that aim, the country has volunteered its specialized tokamak—a one-of-a-kind test reactor containing a swirling ring of plasma—to a global innovation effort. In identifying these materials, Kazakhstan hopes to redefine its legacy: from a source of radioactive harm to a center of nuclear solutions.

“It is gratifying that in Kazakhstan, the science of the peaceful atom is being developed,” Chektybayev tells *C&EN*. “It evokes a feeling of pride, our involvement in the creation of something big and great. Not only for the benefit of a small number of people, but for humanity as a whole.”

## From painful past to hopeful future

Starting in 1949, the Soviet Union conducted more than 450 nuclear tests at the Semipalatinsk Test Site, a swath of open steppe the size of New Jersey. The Soviet government chose the area because of its supposed remoteness.

But the government neglected the Kazakh villagers and nomadic herders who called the region home. These people suffered from the effects of radiation that spread through the air and water, and they continue to experience high rates of cancer and birth defects.

The fallout reached urban environments, too. Some radioactive material made it to the city of Semipalatinsk, now known as Semey, an industrial hub along the Irtysh River less than a 2 h drive to the east of the test site.

**Figure:**
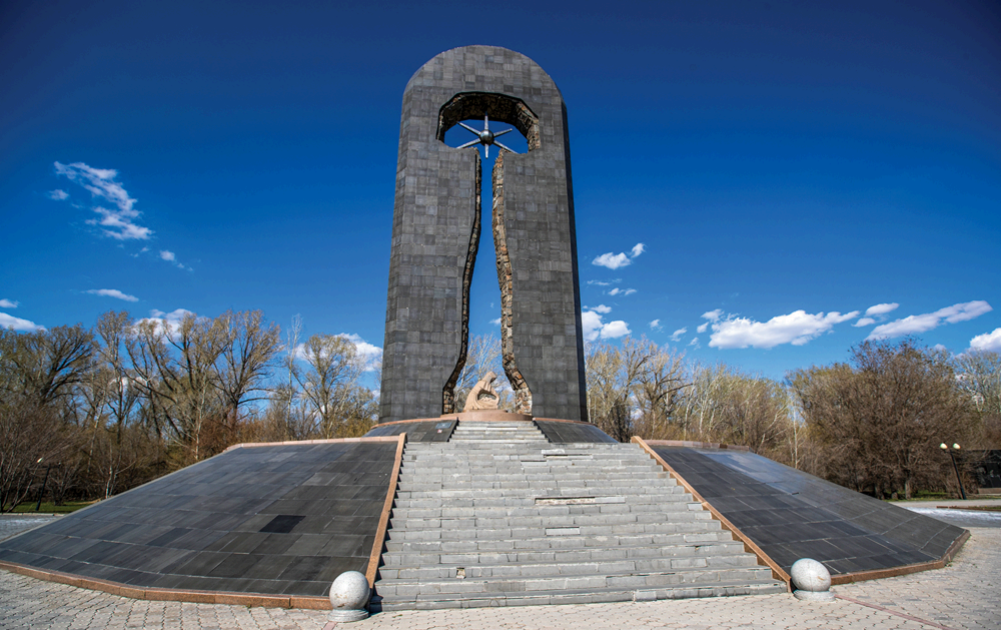
Erected in 2002, the Stronger than Death monument in Semey, Kazakhstan, commemorates those who were killed and those who are still harmed by the nuclear weapon experiments done at the Semipalatinsk Test Site. Credit: Shutterstock.

A national antinuclear movement, Nevada-Semipalatinsk, began in 1989 and pushed the Soviet government to announce a moratorium on testing. In 1991, shortly before the Soviet Union collapsed and Kazakhstan gained independence, the test site was finally closed. The following years were difficult ones for the newly independent country and its scientists, especially given a loss of funding that had previously been provided by the Soviet government in Moscow.

Kazakhstan was left with four research reactors and a cadre of nuclear scientists who knew how to operate them but had little funding and few options for applying their skills within the newly independent country. Fears spread that, lacking work, former Soviet scientists would turn to selling their nuclear know-how and access to materials on the black market.

The fledgling government decided to explore a new path: pivoting away from nuclear weapons and instead pursuing nuclear energy research. It opened the NNC in 1992.

In 2007, the year Chektybayev began working at the NNC, it announced that it would build a tokamak, a machine that generates a powerful magnetic field to confine a high-temperature plasma. One day, the NNC said, it could be used in a practical fusion reactor.

The project was meant to bring Kazakhstan one step closer to joining Iter, an international nuclear fusion research and engineering megaproject that brings together the world’s major industrial powers: the US, the European Union, Russia, China, India, Japan, and South Korea.

The Iter reactor is currently under construction in southern France; it will be the largest fusion reactor in the world on its completion, which is expected sometime in the mid-2030s. Iter’s seven members fund and operate the reactor, but the organization also cooperates with nonmember nations that can offer knowledge or research facilities.

Kazakhstan signed a cooperation agreement with Iter in 2017 on the sidelines of an international clean energy expo hosted in the capital, Astana. In it, the NNC pledged to exchange experts with Iter and provide its tokamak for materials testing.

“This is for our mutual benefit,” says Mario Merola, the deputy head of Iter’s engineering services department and the main liaison between the project and the NNC. “You have countries that are specialized or can offer scientific contributions which are not available in the other member states....and they have access to our knowledge. So they learn a lot from us, and we gain a lot from them.”

## A ‘very rare’ place

Before you can build a fusion reactor, you need to decide what it will be made of. From the beginning, the Kazakhstan tokamak for material testing (KTM) was designed for assessing those possible materials.

Materials in a fusion reactor must withstand several extreme conditions, starting with temperature: the fuel for the reaction, which consists of isotopes of hydrogen known as deuterium and tritium, needs to reach 150 million °C in order to compel the nuclei to fuse together, Merola explains. That’s 10 times the temperature of the sun’s core, and although the plasma is contained using powerful magnets, the inner surface of the reactor that faces the suspended plasma must be able to tolerate high levels of heat and radiation.

The conditions inside the KTM are as close as currently possible to those of the future Iter facility, making it the best place on Earth to test different materials. The prospect is a rare opportunity, Merola says. Although many laboratories can conduct testing under high temperatures, tokamaks also incorporate a magnetic field, as well as plasma that interacts with the material.

**Figure:**
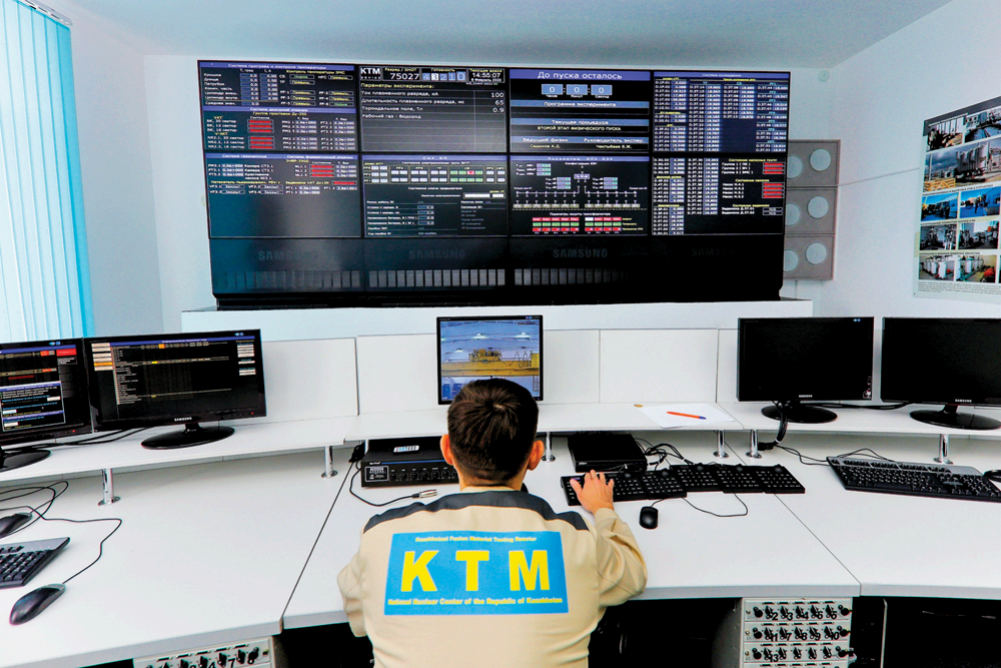
An employee at Kazakhstan’s National Nuclear Center monitors experimental conditions such as temperature and plasma generation at the center’s tokamak. Credit: National Nuclear Center of the Republic of Kazakhstan.

“This is a holistic view,” Merola says. “It’s not just an assessment of a single phenomenon but is an assessment of the phenomena inside the right environment. It’s a very rare feature in the world.”

These plasma experiments are yet to be realized. Although the KTM was able to demonstrate in 2019 that it can generate plasma, the team is conducting additional work to get it ready for full-scale materials testing, according to Erlan Batyrbekov, director general of the NNC.

In the meantime, the NNC has been working with Iter to conduct other kinds of tests. Primarily, the scientists want to see how the materials inside a reactor handle radiation. So far, they have analyzed the effects of radiation on optical sensors and antifriction coatings and have examined load-bearing concrete, which will be used for the base of the Iter tokamak, to see how it holds up under heavy neutron bombardment.

The experiments determined that under these conditions, some impurities in the concrete—namely trace amounts of elements like cesium, europium, samarium, tantalum, and terbium—transform into radioactive isotopes, which could create safety hazards over time.

The NNC has also conducted its own experiments outside its work with Iter, evaluating what other materials could work best inside a future fusion reactor. Of particular interest is finding which material to use in the diverter—the part of the reactor that faces the plasma.

This diverter material will have to solve multiple problems at once. On the one hand, it has to withstand constant pummeling by the incredibly hot plasma. On the other, the diverter surface needs to be able to deteriorate to some degree so that its atoms can provide fuel for the reaction as it runs.

At first glance, stable elements with low atomic numbers seem better suited for the first task. Light materials such as beryllium and carbon don’t react quickly with the deuterium and tritium in the plasma. At the same time, because light elements are highly vulnerable to erosion, plasma can dislodge, or sputter, their atoms from the diverter’s surface.

One compromise might be carbidized tungsten. With its high atomic number and melting point, tungsten doesn’t erode easily, and combining it with carbon makes the material less reactive. The NNC has already conducted some experiments with carbidized tungsten and sees it as a promising material for fusion reactor surfaces.

While that material might solve the resiliency problem, NNC researchers are still working out how they would fuel a reactor. Although deuterium can easily be extracted from many common materials such as seawater and fed into the machine, tritium is radioactive and found only in trace amounts in nature and so must be produced entirely inside the reactor.

This is typically done by coating the diverter with a breeding blanket. Essentially just a layer of lithium, the blanket releases tritium as it gets seared by the tokamak’s plasma. Each lithium atom hit with a neutron breaks into a tritium and a helium atom and feeds the reaction in a self-sustaining loop.

Researchers at the NNC have experimented with using lithium-containing ceramics such as lithium metatitanate. Unlike lithium alone, these materials can withstand high temperatures and radiation exposure over long periods.

The scientists have also tested lithium capillary-porous structures, which Batyrbekov says “combine the advantages of liquid metals”—namely the ability to flow and withstand high heat without cracking—“with the structural stability of a solid matrix.”

## A vision for progress

Kazakhstan’s cooperation agreement with Iter was renewed earlier this year. Batyrbekov says that once the KTM is fully up and running, the NNC plans to test potential materials for the diverter surface. In particular, the center will study how materials interact with plasma as well as watch for erosion, radiation damage, and deposition—the gradual buildup of plasma particles on the tokamak’s inner surface. He envisions eventually creating an international thermonuclear fusion laboratory at the NNC where researchers from different countries can conduct joint experiments in plasma physics.

The NNC is able to do this work because of its unique combination of historical heritage and government investment, Batyrbekov says. Kazakhstan is one of only two lower-income nations to have signed a cooperation agreement with Iter—the other is Thailand, which benefits primarily from knowledge-sharing partnerships with Iter rather than from conducting its own testing. Both Batyrbekov and Chektybayev, who is now the laboratory head at the NNC’s Institute of Atomic Energy, aim to show what countries can do if technology and human capital are channeled toward a project like nuclear fusion—and, hopefully, bring the technology’s perpetual 30-year horizon just a bit closer.

“The creation of the National Nuclear Center in Kurchatov has become a real embodiment of change: from a testing ground to science, from destruction to creation,” Batyrbekov says. “That is why our motto is ‘From national tragedy to national pride.’”

*Diana Kruzman is a freelance contributor to* Chemical & Engineering News *, the independent news publication of the American Chemical Society.*

